# Diagnostic and prognostic significance of systemic alkyl quinolones for *P. aeruginosa* in cystic fibrosis: A longitudinal study

**DOI:** 10.1016/j.jcf.2016.10.005

**Published:** 2017-03

**Authors:** Helen L. Barr, Nigel Halliday, David A. Barrett, Paul Williams, Douglas L. Forrester, Daniel Peckham, Kate Williams, Alan R. Smyth, David Honeybourne, Joanna L. Whitehouse, Edward F. Nash, Jane Dewar, Andrew Clayton, Alan J. Knox, Miguel Cámara, Andrew W. Fogarty

**Affiliations:** a Division of Respiratory Medicine, University of Nottingham, City Hospital Campus, Nottingham, UK; b School of Life Sciences, Centre for Biomolecular Sciences, University of Nottingham, Nottingham, UK; c Centre for Analytical Bioscience, School of Pharmacy, University of Nottingham, Nottingham, UK; d Leeds Adult Cystic Fibrosis Centre, St James's University Hospital, Leeds, UK; e Division of Child Health, Obstetrics and Gynaecology, University of Nottingham, UK; f West Midlands Adult CF Centre, Heart of England, NHS Foundation Trust, Birmingham, UK; g Wolfson Cystic Fibrosis Centre, Department of Respiratory Medicine, Nottingham University Hospitals NHS Trust, Nottingham, UK; h Division of Epidemiology and Public Health, University of Nottingham, Clinical Sciences Building, University of Nottingham, Nottingham, UK

## Abstract

**Background:**

Pulmonary *P. aeruginosa* infection is associated with poor outcomes in cystic fibrosis (CF) and early diagnosis is challenging, particularly in those who are unable to expectorate sputum. Specific *P. aeruginosa* 2-alkyl-4-quinolones are detectable in the sputum, plasma and urine of adults with CF, suggesting that they have potential as biomarkers for *P. aeruginosa* infection.

**Aim:**

To investigate systemic 2-alkyl-4-quinolones as potential biomarkers for pulmonary *P. aeruginosa* infection.

**Methods:**

A multicentre observational study of 176 adults and 68 children with CF. Cross-sectionally, comparisons were made between current *P. aeruginosa* infection using six 2-alkyl-4-quinolones detected in sputum, plasma and urine against hospital microbiological culture results. All participants without *P. aeruginosa* infection at baseline were followed up for one year to determine if 2-alkyl-4-quinolones were early biomarkers of pulmonary *P. aeruginosa* infection.

**Results:**

*Cross-sectional analysis*: the most promising biomarker with the greatest diagnostic accuracy was 2-heptyl-4-hydroxyquinoline (HHQ). In adults, areas under the ROC curves (95% confidence intervals) for HHQ analyses were 0.82 (0.75–0.89) in sputum, 0.76 (0.69–0.82) in plasma and 0.82 (0.77–0.88) in urine. In children, the corresponding values for HHQ analyses were 0.88 (0.77–0.99) in plasma and 0.83 (0.68–0.97) in urine.

*Longitudinal analysis:* Ten adults and six children had a new positive respiratory culture for *P. aeruginosa* in follow-up. A positive plasma HHQ test at baseline was significantly associated with a new positive culture for *P. aeruginosa* in both adults and children in follow-up (odds ratio (OR) = 6.67;-95% CI:-1.48–30.1;-*p* = 0.01 and OR = 70; 95% CI: 5–956;-*p* < 0.001 respectively).

**Conclusions:**

AQs measured in sputum, plasma and urine may be used to diagnose current infection with *P. aeruginosa* in adults and children with CF. These preliminary data show that plasma HHQ may have potential as an early biomarker of pulmonary *P. aeruginosa*. Further studies are necessary to evaluate if HHQ could be used in clinical practice to aid early diagnosis of *P. aeruginosa* infection in the future.

## 1 Introduction

Chronic pulmonary infection with *Pseudomonas aeruginosa* in patients with cystic fibrosis (CF) is an important risk factor contributing to recurrent hospital admissions [1] and increased mortality [1]. The prevalence of *P. aeruginosa* infection in CF increases with age, with approximately two thirds of adults either intermittently or chronically colonised with this bacterium [2]. In the early stages of infection, *P. aeruginosa* may be eradicated from the lungs with targeted antimicrobial therapy [3], [4] which positively affects the long term course of the disease [5].

There is a clinical need for better early markers for pulmonary infection with *P. aeruginosa* to facilitate early intervention with eradication therapy. In current clinical practice, the main technique used to diagnose pulmonary *P. aeruginosa* infection is conventional microbiological culture of respiratory specimens [6]. Spontaneous sputum is quick and simple to culture and is widely assumed to accurately diagnose common lower airway pathogens [7]. However, early diagnosis of pulmonary *P. aeruginosa* infection is more challenging in those who are unable to expectorate sputum, such as young children and adults with well maintained lung function. In these circumstances, diagnostic options include sampling from the upper airways using cough swabs, which have a low sensitivity and may miss lower airways infection [8], or the use of more invasive techniques such as induced sputum [9] or bronchoalveolar lavage [9]. More recently, serological antibody tests against *P. aeruginosa* have been used to aid diagnosis of infection [10], [11], [12], [13], [14], [15], [16], [17]. Studies have demonstrated that these antibodies can be used as markers of chronic infection with *P. aeruginosa* [17], but their ability to predict early infection with *P. aeruginosa* has so far been relatively disappointing [11], [14], [17], [18]. Finally, culture independent techniques using molecular primer sequencing are increasingly being used in a research setting to diagnose infection in the lower airways [19], however these still require direct airway samples and are subjected to false positives due to sample contamination and the high sensitivity of these techniques.

In this study, we use novel LC–MS/MS methodology [20] to directly measure specific quorum sensing signal molecules (alkyl quinolones, AQs) in the systemic circulation of patients with CF. These AQs regulate virulence factor production [21], possess anti-staphylococcal properties [22], and have been detected in infants with CF [23], suggesting that they may play a role in the early stages of *P. aeruginosa* infection. The aim of this study was to assess the diagnostic and prognostic potential of plasma and urinary AQs as biomarkers for pulmonary *P. aeruginosa* infection in patients with CF.

## 2 Methods

### 2.1 Participants

Patients were recruited from three adult CF centres (Nottingham University hospitals NHS Trust, Heart of England NHS Foundation Trust and Leeds Teaching Hospitals NHS Trust) and one paediatric CF centre (Nottingham University hospitals NHS Trust). Inclusion criteria were a diagnosis of CF and exclusion criteria were any previous isolation of *Burkholderia cepacia* complex in sputum. Control participants with no known respiratory conditions and no history of *P. aeruginosa* infection were invited to participate in the study. Informed written consent was obtained from all participants or their legal representative and the study was approved by the Nottingham Research Ethics Committee 1 (09/H0407/1).

### 2.2 Study design

Adults with CF were recruited at routine CF clinic appointments and were clinically stable at the study visit, having not experienced a pulmonary exacerbation requiring intravenous (IV) antibiotics in the previous 4 weeks. Spontaneous sputum, 8 ml blood and 25 ml urine samples were obtained. Pulmonary function tests were performed according to standardised criteria [24] and validated CFQ quality of life questionnaires were completed [25].

Children with CF were recruited from paediatric CF clinics and samples of plasma and urine were obtained at annual assessment. Sputum collection was not requested from children.

A control population of healthy individuals was recruited by poster advertisement and provided venous blood and urine samples.

### 2.3 Sputum, blood and urine processing

Spontaneous sputum samples were collected according to standard guidelines [26], [27]. Sputum plugs were harvested and processed for quantitative QSSM analysis [28] and differential cell counts [26], [27]. Sputum plugs for quantitative microbiological analysis were mixed with an equal volume of dithiothreitol and diluted with 0.9% w/v saline and 100 μl of × 10^− 2^ and × 10^− 4^ solutions were plated on blood and pseudomonas isolation agar (Difco; BD, Sparks, MD, USA). The plates were incubated at 37 °C and colony counts were performed daily between 24 and 72 h, until maximal growth was achieved. Venous blood samples were centrifuged at 1000*g* for 15 min at 4 °C and plasma was separated and snap frozen in liquid nitrogen. All biological samples were stored on ice and frozen at − 80 °C within 4 h.

### 2.4 AQ analysis

Clinical samples for AQ analysis were analysed using LC–MS/MS [20]. Measurement of AQ levels in clinical samples was performed by comparison of extracts with calibration standards. Further details of the sample preparation, LC–MS/MS analysis and quantification can be found in the online data supplement (S1). A total of six AQs were studied: HHQ (2-heptyl-4-hydroxyquinoline), NHQ (2-nonyl-4-hydroxyquinoline), C7-PQS (2-heptyl-3-hydroxy-4(1*H*)-quinolone), C9-PQS (2-nonyl-3-hydroxy-4(1*H*)-quinolone), HQNO (2-heptyl-4-hydroxyquinoline-*N*-oxide) and NQNO (2-nonyl-4-hydroxyquinoline-*N*-oxide) For 2-alkyl-4(1*H*)-quinolone (AQ) and 2-alkyl-4-hydroxyquinoline *N*-oxide molecules, a previously accepted nomenclature has been adopted [29]. A positive AQ test was defined as a measured AQ concentration greater or equal to the lower limit of quantification in the media of interest obtained by LC–MS/MS (see online supplement, E1).

### 2.5 Diagnosis of *P. aeruginosa*

Current hospital microbiological culture results were used as the reference standard for a diagnosis of *P. aeruginosa* and were obtained from cough swabs, spontaneous sputum cultures or bronchoalveolar lavage (BAL) using standard protocols [30]. Patients were categorised into four groups using the Leeds criteria for *P. aeruginosa* [31]. The four groups were: ‘chronic’ (defined as ≥ 50% of samples positive for *P. aeruginosa* in the last 12 months); ‘intermittent’ (defined as < 50% of samples positive for *P. aeruginosa* in the last 12 months); ‘free’ (defined as previous *P. aeruginosa* infection but not isolated in the last 12 months) and ‘never’ (defined as those who had not previously isolated *P. aeruginosa*).

### 2.6 Statistical analysis

AQ concentrations in sputum, plasma and urine were compared using scatter graphs and Spearman rank correlation co-efficients. Each of the six AQs were compared to hospital microbiological culture results in both adults and children with CF. Sensitivity, specificity, positive predictive values and negative predictive values were calculated for each AQ in sputum, plasma and urine. Non-parametric receiver operator characteristic (ROC) curves [30] and areas under the curves (AUC) were used to compare each AQ to current hospital culture results. Comparisons between *P. aeruginosa* groups were assessed using Kruskal-Wallis testing. In the longitudinal analysis, logistic regression was used to compare the presence or absence of AQs in plasma and urine of adults and children who had no evidence of *P. aeruginosa* infection in the previous year with hospital respiratory culture results in the subsequent 12 months.

The association between AQ levels in plasma and clinical variables of interest was explored using Spearman rank correlation co-efficients or Mann Whitney U tests. These explanatory variables were age, gender, forced expiratory volume in 1 s (FEV_1_) percent predicted, quantitative load of *P. aeruginosa* measured on pseudomonas isolation agar, sputum neutrophil concentrations and the use of maintenance azithromycin and nebulised antibiotics. All data were analysed using STATA 11 statistical software (Texas, USA).

## 3 Results

244 patients with CF were recruited, 176 adults and 68 children. Clinical characteristics, *P. aeruginosa* status of the participants and hospital trust recruited from are shown in Table 1. In addition, 22 control participants were recruited from the general population (11 males, median age = 31.7 years).

### 3.1 AQ concentrations in sputum, plasma and urine are positively correlated between the biofluids

AQ concentrations were positively correlated between sputum, plasma and urine with the strongest correlations between sputum and plasma concentrations of HQNO (r = 0.84, *p* < 0.0001) and between sputum and urine levels of HHQ (r = 0.79, *p* < 0.0001; online supplement E2).

### 3.2 The use of AQs in sputum, plasma and urine to diagnose current pulmonary *P. aeruginosa* infection

To evaluate the potential of AQs to diagnose current *P. aeruginosa* infection, AQ results were compared to current hospital respiratory culture results. In adults, the hospital culture results were obtained from 136 spontaneous sputum samples and 40 cough swabs. In children, 46 samples were cough swabs, 20 samples were spontaneous sputum and 2 samples were obtained from BAL washings. Sensitivity and specificity data for all six AQs are shown in the online supplement (E3 and E4). Three patients had a new positive respiratory culture for *P. aeruginosa* at the baseline visit, having not isolated this bacterium in the preceding year, and AQs were detectable in all 3 patients (online supplement E5).

Area under curve (AUC) results for each of the six AQs in each biofluid are summarised in Table 2.

**Fig. 1:**
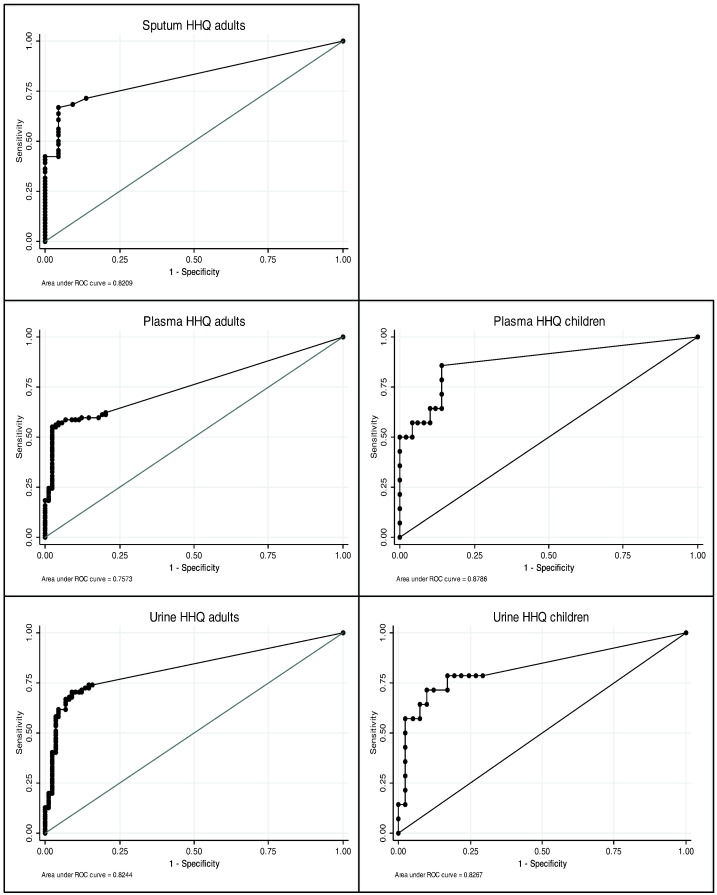
Receiver operator curves for 2-heptyl-4-hydroxyquinoline (HHQ) in sputum, plasma and urine in adults and children with cystic fibrosis compared to hospital microbiological culture results.

Of the six AQs studied, the most promising biomarkers (with the highest AUCs and therefore greatest diagnostic accuracy) were HHQ (Fig. 1) and HQNO, and further analyses focused on these two molecules. In adults, the sensitivity and specificity of HHQ in plasma were 62% (95% CI: 51–73) and 80% (95% CI: 70–88) respectively and the corresponding values in urine were 74% (95% CI: 63–83) and 84% (95% CI: 75–91) respectively (Table 3). In children, the presence of HHQ in plasma had a sensitivity of 86% (95% CI: 57–98) and specificity of 86% (95% CI: 73–94), and the corresponding values in urine were 79% (95% CI: 49–95) and 71% (95% CI: 55–84) respectively (Table 3). The prevalence of HHQ and HQNO in each media is shown in the on line supplement (E6).

### 3.3 Relationship between plasma HHQ and HQNO with *P. aeruginosa* infection status

**Fig. 2:**
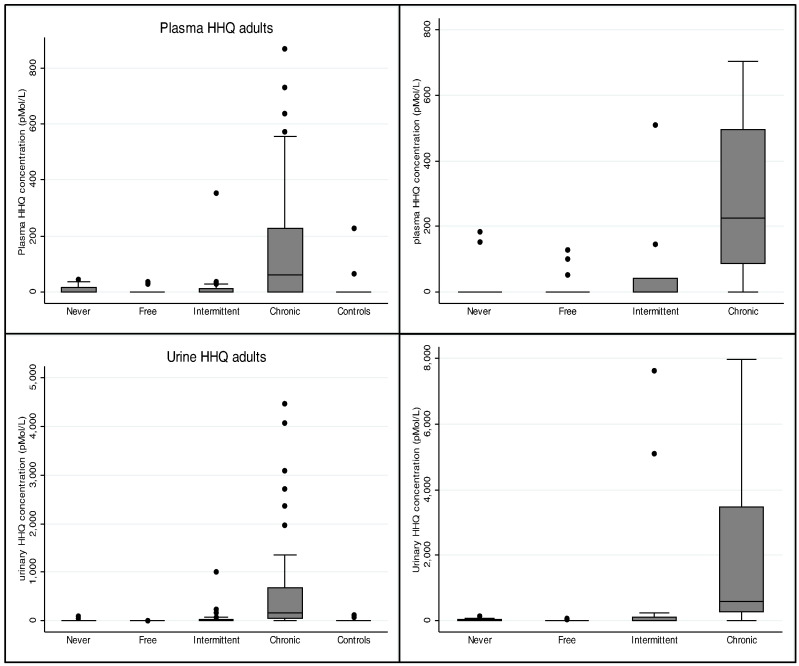
2-heptyl-4-hydroxyquinoline (HHQ) concentration as a function of *P. aeruginosa* status in adults and children with cystic fibrosis and adult control participants measured in the plasma and urine. Prevalence of HHQ in plasma above the lower limit of quantification using LC–MS/MS stratified by *P. aeruginosa* status in adults: 25% (5/20) categorised as ‘never’, 18% (7/38) categorised as ‘free’, 27% (8/30) categorised as ‘intermittent’ and 60% (50/84) categorised as ‘chronic’ and 9% (2/22) in healthy controls. In children, the prevalence of HHQ in plasma, stratified by *P. aeruginosa* status was: 11% (2/18) in the ‘never’ category, 165 (4/25) in ‘free’, 30% (3/10) in ‘intermittent’, 91% (10/11) in ‘chronic’ category. HHQ was detectable in the plasma of 3 controls and urine of 4 controls.

Clinical samples from all patients with CF, stratified by *P. aeruginosa* status were analysed, focusing on the two most promising biomarkers, HHQ and HQNO. There were significant differences in AQ concentrations between samples obtained from adults with different categories of *P. aeruginosa* infection and the control participants in all biofluids (Kruskal-Wallis test: *p* ≤ 0.003 for both HHQ and HQNO in sputum, plasma and urine; Fig. 2). Similar results were observed in plasma and urine samples obtained from children with CF (Fig. 2, *p* < 0.02).

### 3.4 Relationship between plasma HHQ and HQNO and clinical status in adults (n = 176)

Overall, HHQ and HQNO levels in the plasma of adults were negatively correlated with percent-predicted FEV_1_ (Spearman rank correlation coefficients, r = − 0.19; *p* = 0.01 and r = − 0.25; *p* = 0.001 respectively) and this association was not significantly affected by adjustment for age. HHQ in plasma was positively correlated with quantitative load of *P. aeruginosa* in sputum (Spearman rank correlation co-efficient: r = 0.28, *p* = 0.02) and negatively correlated with the use of maintenance azithromycin, with higher values in those who were receiving maintenance azithromycin therapy compared to those who were not (Mann–Whitney U test: *p* = 0.004). No significant associations were observed between plasma HQNO and *P. aeruginosa* load or plasma HQNO and the use of azithromycin. There were no significant associations between plasma HHQ and HQNO levels in adults and sputum neutrophil concentration, age, gender, the use of maintenance nebulised antibiotics or respiratory quality of life responses (data not shown).

### 3.5 Longitudinal analysis: use of plasma and urinary AQs to detect early *P. aeruginosa* infection

In the year following QSSM testing, a new positive respiratory tract culture for *P. aeruginosa* was reported in and 6 (14%) children and 10 (18%) adults who were categorised as ‘free’ or ‘never’ having isolated *P. aeruginosa* at baseline (Table 4 and online supplement E7).

The presence of HHQ in plasma at baseline was significantly associated with a new positive respiratory tract *P. aeruginosa* culture in the following year in both adults and children with CF (OR = 6.67; 95% CI 1.48 to 30.1; *p* = 0.01 and OR = 70; 95% CI: 5 to 956; *p* < 0.001 respectively; Table 4 and online supplement E7).

The presence of HHQ in urine or HQNO in plasma or urine at baseline was not significantly associated with a new positive *P. aeruginosa* respiratory tract culture in the following year in children or adults (online supplement E7).

## 4 Discussion

This is the first study to test the diagnostic and prognostic significance of systemic AQ quorum sensing signal molecules as biomarkers for pulmonary *P. aeruginosa* in adults and children with CF. Of the QSSMs measured, the diagnostic accuracy of HHQ and HQNO were highest, suggesting these molecules have potential as biomarkers of infection with *P. aeruginosa*. The follow up data also permitted identification of HHQ in plasma as a potential early biomarker of *P. aeruginosa*. The consistency in these observations in both adults and children with CF suggests that this finding may be generalizable across all age groups. If externally validated, these findings would be particularly important for young children with CF, who stand to benefit the most from an early diagnostic test for *P. aeruginosa.* This study also showed that (1) higher concentrations of HHQ in plasma were associated with lower percent-predicted FEV_1_ and higher bacterial load and (2) systemic AQs concentrations were elevated in patients with newly acquired *P. aeruginosa* infection, suggesting that these molecules may be associated with an adverse prognosis. These data also support our previous findings that systemic levels of some AQs are elevated during pulmonary exacerbations in adults with CF [33].

Strengths of the study include the large study population, who were recruited from three specialist CF centres, with a wide range of disease severities, which may be generalizable to the broader UK CF population. The direct comparison of sputum, plasma and urinary AQ levels in 87 adults demonstrated that these were highly correlated, providing confidence that the lower airways were the source of the AQs. We used routine hospital culture results as our reference standard for *P. aeruginosa*, thus comparing our test with current clinical practice, and this was a necessary part of the study design. However, this may underestimate the true value of the new tests, as the diagnostic accuracy of both cough swabs and sputum cultures is lower than BAL [6], [32], which is considered the ‘gold standard’ for defining lower airway microbiology.

There are several factors that require consideration when interpreting these data. Firstly, multiple hypotheses were tested and some of the associations observed may have occurred by chance, although as most of the *p* values are small, this is unlikely. Secondly, interpreting the clinical significance of low levels of AQs is challenging. Low concentrations of several AQs were detected in some adults who were classified as ‘free’ or ‘never’ having had *P. aeruginosa* infection and also in a minority of the control population. This may represent environmental exposure resulting in subclinical infection and further investigation into these findings is warranted. Thirdly, the numbers of patients who were negative for *P. aeruginosa* at recruitment and subsequently developed infection was small. This is reflected in the wide confidence intervals of the ORs, which should be taken into account when interpreting these data. Fourthly, we observed that patients with higher AQ concentrations were more likely to be currently prescribed oral azithromycin. There is currently no evidence that azithromycin affects AQ production, although it inhibits the in vitro production of some quorum sensing signal molecules from the *N*-acyl-L-homoserine lactone class [34]. We suggest that the use of azithromycin may be a confounding factor in this study, as patients with chronic *P. aeruginosa* infection were more likely to be prescribed azithromycin and chronic *P. aeruginosa* infection was also associated with higher AQ levels. In cross sectional analysis, the use of maintenance nebulised antibiotics was not associated with differences in AQ concentrations in plasma and urine. However, further studies would be necessary to assess the impact of antibiotic therapy on AQ levels at an individual level. Fifthly, the predictive values for AQ tests should be interpreted in the context of disease prevalence, as a higher disease prevalence is associated with higher positive predictive values (PPVs) and lower negative predictive values (NPVs) [35]. Thus, AQ testing in children be used to confidently rule out infection (high NPV) but there is a higher false positive rate (low PPV) compared to the adult population, who had a higher prevalence of *P. aeruginosa* infection.

Recently, serological antibody tests have been used to aid the diagnosis of *P. aeruginosa* infection. Studies report varying accuracies for serological tests, depending on the antigens tested, the defined cut points, patient cohort and the test standard used [11], [14], [17], [36]. Whilst direct comparisons between these studies are not possible, the diagnostic accuracy of AQ tests in this study is broadly similar to serological test results. However, there are some limitations of serological antigen testing. Serological tests rely on detecting the host immune response to infection and titres can be affected by co-infection in the lungs [37] and by the concurrent use of antibiotics [13], [16], [37], [38] or oral steroids [39]. Potential advantages of AQ testing compared to serological testing include the direct detection of bacterial products themselves and the ability to detect these molecules in the urine, which would allow non-invasive testing in children.

We acknowledge that this study represents an early step to determine if AQs could aid clinical decision-making in CF in future. One area for further investigation includes the testing of AQs in control populations, in particular, the paediatric population and also in patients with *Burkholderia cepacia* complex. Another challenge is determining the clinical significance of low levels of AQs. This is particularly important, as low concentrations of AQs were detectable in the plasma and urine of a minority of the control population. Understanding the role of AQs in early infection with *P. aeruginosa* in the CF population is another area that deserves attention, as in vivo data are limited [40], [41] and most of our current knowledge comes from animal models or in vitro studies [20]. Other areas that warrant investigation are the effects of antibiotics, including eradication therapy, on systemic AQ levels and understanding the metabolism and excretion of AQs. Further steps include an external validation study, optimization of cut points and comparisons of AQ tests to other tests for *P. aeruginosa* used in clinical practice such as BAL, serological tests and PCR. Finally, to bring this test to routine clinical practice would also require development of a widely available laboratory or point of care test, such as a biosensor for AQs [42], which could be developed into a quick and cost effective bedside test for *P. aeruginosa*.

In summary, these data suggest that AQs can be used to determine current infection with *P. aeruginosa*. Limited data also show that HHQ detected in the plasma may be an early biomarker for pulmonary *P. aeruginosa* infection. If validated, this finding would be particularly relevant in young children, where diagnosis of pulmonary infection with *P. aeruginosa* is challenging and early detection of *P. aeruginosa* is sought to initiate timely eradication therapy. The high negative predictive levels of plasma and urinary AQs in children suggest that these tests may be useful in ruling out *P. aeruginosa* infection, thus supporting robust infection control measures and risk stratification of patients in CF clinics.

## Author statement

HLB/MC/DB/NH/DLF/AS/AWF contributed to the concept, design, acquisition of data, interpretation of data, drafting of manuscript and approval of final version of manuscript. AJK/PW contributed to the concept, design, interpretation of data, drafting of manuscript and approval of final version of manuscript. KW/DP/DH/JW/EN/AC contributed to the acquisition of data, interpretation of data, drafting of manuscript and approval of final version of manuscript.

## Competing interests

The University of Nottingham has a patent pending for the use of alkyl quinolones as biomarkers for *P. aeruginosa* infection (PCT/GB2014/051458).

## Funding

Medical Research Council (G0801558); Doug Forrester is funded by the Wellcome Trust, fellow number: WT088614.

## Figures and Tables

**Table 1:** Clinical characteristics and *P. aeruginosa* status of participants with cystic fibrosis.

Variable	Adults	Children
Hospital		
Nottingham University Hospitals NHS Trust	64	68
Heart of England NHS Foundation Trust	76	
Leeds teaching Hospitals NHS Trust	36	
Age in years: median (range)	27.8 (17.7 to 65.2) n = 176	9.6 (0.8 to 17.0) n = 68
Gender, males (%)	104 (59)	31 (46)
FEV_1_ percent predicted: mean (range)	65 (17 to 110) n = 166	79 (41 to 121) n = 35
Absolute FEV_1_ in L: mean (range)	2.40 (0.71 to 4.78) n = 165	1.70 (0.60 to 3.69) n = 34
*P. aeruginosa* status at baseline visit: n(%)		
Never	20 (11)	19 (28)
Free	39 (22)	25 (37)
Intermittent	30 (17)	13 (19)
Chronic	86 (49) n = 175⁎	11 (16) n = 68
Maintenance treatment:		
Azithromycin	105 (60)	35 (51)
Cycled nebulised Colomycin	83 (48)	11 (16)
Cycled nebulised Tobramycin	38 (22)	3 (4)
Number of patients with a positive respiratory culture for *P. aeruginosa* at baseline in the hospital laboratory, n (%)	84 (48%)^	14 (21%)

n = number of participants with data available.

*P. aeruginosa* status of patients defined by the Leeds criteria [30].

⁎ 1 patient had no available respiratory samples in the previous 12 months and *P. aeruginosa* status could therefore not be assessed.

^ Data missing for 2 patients.

**Table 2:** Area under receiver operating characteristic curves for six quorum sensing signalling molecules in biological samples from patients with cystic fibrosis compared to current hospital respiratory culture results.

2-alkyl-4-quinolone tested	Adults			Children	
	Sputum n = 88	Plasma n = 171	Urine n = 173	Plasma n = 64	Urine n = 55
	AUC (95% CI)	AUC (95% CI)	AUC (95% CI)	AUC (95% CI)	AUC (95% CI)
HHQ	0.82 (0.75–0.89)	0.76 (0.69–0.82)	0.82 (0.77–0.88)	0.88 (0.77–0.99)	0.83 (0.68–0.97)
HQNO	0.86 (0.79–0.93)	0.73 (0.67–0.78)	0.73 (0.67–0.79)	0.78 (0.64–0.92)	0.83 (0.70–0.96)
NHQ	0.82 (0.75–0.90)	0.58 (0.51–0.64)	0.63 (0.56–0.70)	0.65 (0.52–0.78)	0.69 (0.52–0.85)
NQNO	0.84 (0.76–0.91)	0.67 (0.62–0.73)	0.56 (0.52–0.59)	0.71 (0.57–0.84)	0.62 (0.49–0.74)
C7-PQS	0.71 (0.61–0.81)	0.66 (0.60–0.71)	0.66 (0.61–0.72)	0.71 (0.58–0.85)	0.72 (0.57–0.87)
C9-PQS	0.73 (0.62–0.85)	0.55 (0.51–0.58)	0.53 (0.49–0.57)	0.63 (0.51–0.75)	0.61 (0.47–0.75)
Prevalence of current PA infection using respiratory culture results (%)	75	48	49	22	25

AUC = Area under Receiver Operating Characteristics curve.

*PA* *=* *Pseudomonas aeruginosa.*

HHQ = 2-heptyl-4-hydroxyquinoline.

HQNO = 2-heptyl-4-hydroxyquinoline-*N*-oxide.

NHQ = 2-nonyl-4-hydroxyquinoline.

NQNO = 2-nonyl-4-hydroxyquinoline-*N*-oxide.

C7-PQS = 2-heptyl-3-hydroxy-4(1*H*)-quinolone.

C9-PQS = 2-nonyl-3-hydroxy-4(1*H*)-quinolone.

n = number of samples available for analysis.

**Table 3:** Evaluation of 2-heptyl-4-hydroxyquinoline (HHQ) for the diagnosis of *P. aeruginosa* compared to standard microbiological culture in adults and children.

Test (95% CI)	Adults			Children	
	Sputum n = 88	Plasma n = 171	Urine n = 173	Plasma n = 64	Urine n = 55
Sensitivity, % (95% CI)	71 (59–82)	62 (51–73)	74 (63–83)	86 (57–98)	79 (49–95)
Specificity, % (95% CI)	86 (65–97)	80 (70–88)	84 (75–91)	86 (73–94)	71 (55–84)
PPV, % (95% CI)	94 (84–99)	74 (62–84)	82 (71–90)	63 (38–84)	48 (27–69)
NPV, % (95% CI)	50 (33–67)	70 (60–78)	77 (68–85)	96 (85–99)	91 (75–98)
Prevalence of PA using respiratory cultures results, %	75	48	49	22	25

PPV = positive predictive value.

NPV = negative predictive value.

PA = *Pseudomonas aeruginosa.*

A positive test was defined as an HHQ concentration greater or equal to the lower level of quantification in the sample type (see online supplement S1).

n = number of samples available for analysis.

**Table 4:** Relationships between baseline plasma 2-heptyl-4-hydroxyquinoline (HHQ) and respiratory culture results for *P. aeruginosa* in the 12 month follow up period for participants with CF who were classified as ‘free’ or ‘never’ having isolated *P. aeruginosa* at baseline^.

*P. aeruginosa* culture ‘free’ or ‘never’ at baseline^	Plasma HHQ result at baseline visit#	Remained *P. aeruginosa* culture negative during follow up	New positive culture for *P. aeruginosa* during follow up
Adults n = 56⁎	HHQ negative	40	5
	HHQ positive	6	5
Children n = 42♦	HHQ negative	35	2
	HHQ positive	1	4

n = number of samples available for analysis.

# A positive test was defined as an HHQ concentration greater or equal to the lower level of quantification (see online supplement S1).

^ *P. aeruginosa* status of patients defined by the Leeds criteria [32].

⁎ 3 adults had no respiratory cultures reported by the hospital laboratory during 1-year follow up.

♦ 2 children did not provide plasma samples at the baseline visit.

## Appendix A Supplementary data

Supplementary material Image 1

## References

[1] Emerson J., Rosenfeld M., McNamara S., Ramsey B., Gibson R.L. (2002). *Pseudomonas aeruginosa* and other predictors of mortality and morbidity in young children with cystic fibrosis. Pediatr Pulmonol.

[2] Smith D.L., Freeman W., Cayton R.M., Stableforth D.E. (1994). Nocturnal hypoxaemia in cystic fibrosis: relationship to pulmonary function tests. Respir Med.

[3] Valerius N.H., Koch C., Hoiby N. (1991). Prevention of chronic *Pseudomonas aeruginosa* colonisation in cystic fibrosis by early treatment. Lancet.

[4] Ratjen F., Doring G., Nikolaizik W.H. (2001). Effect of inhaled tobramycin on early *Pseudomonas aeruginosa* colonisation in patients with cystic fibrosis. Lancet.

[5] (2011). Cystic fibrosis trust. Standards for the clinical care of children and adults with cystic fibrosis in the UK.

[6] Gilljam H., Malmborg A.S., Strandvik B. (1986). Conformity of bacterial growth in sputum and contamination free endobronchial samples in patients with cystic fibrosis. Thorax.

[7] Rosenfeld M., Emerson J., Accurso F., Armstrong D., Castile R., Grimwood K. (1999). Diagnostic accuracy of oropharyngeal cultures in infants and young children with cystic fibrosis. Pediatr Pulmonol.

[8] De Boeck K., Alifier M., Vandeputte S. (2000). Sputum induction in young cystic fibrosis patients. Eur Respir J.

[9] Wainwright C.E., Vidmar S., Armstrong D.S., Byrnes C.A., Carlin J.B., Cheney J. (2011). Effect of bronchoalveolar lavage-directed therapy on *Pseudomonas aeruginosa* infection and structural lung injury in children with cystic fibrosis: a randomized trial. JAMA.

[10] Pedersen S.S., Espersen F., Hoiby N. (1987). Diagnosis of chronic Pseudomonas aeruginosa infection in cystic fibrosis by enzyme-linked immunosorbent assay. J Clin Microbiol.

[11] Alton E.W., Stern M., Farley R., Jaffe A., Chadwick S.L., Phillips J. (1999). Cationic lipid-mediated CFTR gene transfer to the lungs and nose of patients with cystic fibrosis: a double-blind placebo-controlled trial. Lancet.

[12] Xu J., Moore J.E., Murphy P.G., Millar B.C., Elborn J.S. (2004). Early detection of *Pseudomonas aeruginosa*–comparison of conventional versus molecular (PCR) detection directly from adult patients with cystic fibrosis (CF). Ann Clin Microbiol Antimicrob.

[13] Burns J.L., Gibson R.L., McNamara S., Yim D., Emerson J., Rosenfeld M. (2001). Longitudinal assessment of *Pseudomonas aeruginosa* in young children with cystic fibrosis. J Infect Dis.

[14] Cohn L.A., Weber A., Phillips T., Lory S., Kaplan M., Smith A. (2001). *Pseudomonas aeruginosa* infection of respiratory epithelium in a cystic fibrosis xenograft model. J Infect Dis.

[15] Ramsey B.W., Pepe M.S., Quan J.M., Otto K.L., Montgomery A.B., Williams-Warren J. (1999). Intermittent administration of inhaled tobramycin in patients with cystic fibrosis. Cystic fibrosis inhaled tobramycin study group. N Engl J Med.

[16] Ratjen F., Walter H., Haug M., Meisner C., Grasemann H., Doring G. (2007). Diagnostic value of serum antibodies in early *Pseudomonas aeruginosa* infection in cystic fibrosis patients. Pediatr Pulmonol.

[17] Ling B.N., Zuckerman J.B., Lin C., Harte B.J., McNulty K.A., Smith P.R. (1997). Expression of the cystic fibrosis phenotype in a renal amphibian epithelial cell line. J Biol Chem.

[18] Smith S.N., Delaney S.J., Dorin J.R., Farley R., Geddes D.M., Porteous D.J. (1998). Effect of IBMX and alkaline phosphatase inhibitors on Cl- secretion in G551D cystic fibrosis mutant mice. Am J Physiol.

[19] Ramsey B.W., Davies J., McElvaney N.G., Tullis E., Bell S.C., Drevinek P. (2011). A CFTR potentiator in patients with cystic fibrosis and the G551D mutation. N Engl J Med.

[20] Heeb S., Fletcher M.P., Chhabra S.R., Diggle S.P., Williams P., Camara M. (2011). Quinolones: from antibiotics to autoinducers. FEMS Microbiol Rev.

[21] Machan Z.A., Taylor G.W., Pitt T.L., Cole P.J., Wilson R. (1992). 2-Heptyl-4-hydroxyquinoline N-oxide, an antistaphylococcal agent produced by *Pseudomonas aeruginosa*. J Antimicrob Chemother.

[22] Diggle S.P., Matthijs S., Wright V.J., Fletcher M.P., Chhabra S.R., Lamont I.L. (2007). The *Pseudomonas aeruginosa* 4-quinolone signal molecules HHQ and PQS play multifunctional roles in quorum sensing and iron entrapment. Chem Biol.

[23] Miller M.R., Crapo R., Hankinson J., Brusasco V., Burgos F., Casaburi R. (2005). General considerations for lung function testing. Eur Respir J.

[24] Quittner A.L., Buu A., Messer M.A., Modi A.C., Watrous M. (2005). Development and validation of the cystic fibrosis questionnaire in the United States: a health-related quality-of-life measure for cystic fibrosis. Chest.

[25] Standards unit PHE. UK standards for microbiology investigations.: Public Health England 2014. https://http://www.gov.uk/government/uploads/system/uploads/attachment_data/file/343994/B_57i2.5.pdf.

[26] Ortori C.A., Dubern J.F., Chhabra S.R., Camara M., Hardie K., Williams P. (2011). Simultaneous quantitative profiling of N-acyl-L-homoserine lactone and 2-alkyl-4(1H)-quinolone families of quorum-sensing signaling molecules using LC–MS/MS. Anal Bioanal Chem.

[27] Ortori C.A., Atkinson S., Chhabra S.R., Camara M., Williams P., Barrett D.A. (2007). Comprehensive profiling of N-acylhomoserine lactones produced by Yersinia pseudotuberculosis using liquid chromatography coupled to hybrid quadrupole-linear ion trap mass spectrometry. Anal Bioanal Chem.

[28] Pavord I.D., Pizzichini M.M., Pizzichini E., Hargreave F.E. (1997). The use of induced sputum to investigate airway inflammation. Thorax.

[29] Cystic Fibrosis Trust. Laboratory standards for processing microbioloigcal samples from people with cystic fibrosis2010. https://http:/www.cysticfibrosis.org.uk/media/82034/CD_Laboratory_Standards_Sep_10.pdf.

[30] Lee T.W., Brownlee K.G., Conway S.P., Denton M., Littlewood J.M. (2003). Evaluation of a new definition for chronic Pseudomonas Aeruginosa infection in cystic fibrosis patients. J Cyst Fibros.

[31] DeLong E.R., DeLong D.M., Clarke-Pearson D.L. (1988). Comparing the areas under two or more correlated receiver operating characteristic curves: a nonparametric approach. Biometrics.

[32] Equi A.C., Pike S.E., Davies J., Bush A. (2001). Use of cough swabs in a cystic fibrosis clinic. Arch Dis Child.

[33] Bala A., Kumar R., Harjai K. (2011). Inhibition of quorum sensing in *Pseudomonas aeruginosa* by azithromycin and its effectiveness in urinary tract infections. J Med Microbiol.

[34] Fletcher M.P., Diggle S.P., Crusz S.A., Chhabra S.R., Camara M., Williams P. (2007). A dual biosensor for 2-alkyl-4-quinolone quorum-sensing signal molecules. Environ Microbiol.

[35] Brenner H., Gefeller O. (1997). Variation of sensitivity, specificity, likelihood ratios and predictive values with disease prevalence. Stat Med.

[36] Donnelly L.F., MacFall J.R., McAdams H.P., Majure J.M., Smith J., Frush D.P. (1999). Cystic fibrosis: combined hyperpolarized 3He-enhanced and conventional proton MR imaging in the lung--preliminary observations. Radiology.

[37] Granstrom M., Ericsson A., Strandvik B., Wretlind B., Pavlovskis O.R., Berka R. (1984). Relation between antibody response to *Pseudomonas aeruginosa* exoproteins and colonization/infection in patients with cystic fibrosis. Acta Paediatr Scand.

[38] Brett M.M., Ghoneim A.T., Littlewood J.M. (1987). Serum IgG antibodies in patients with cystic fibrosis with early *Pseudomonas aeruginosa* infection. Arch Dis Child.

[39] Brett M.M., Ghoneim A.T., Littlewood J.M. (1988). Prediction and diagnosis of early *Pseudomonas aeruginosa* infection in cystic fibrosis: a follow-up study. J Clin Microbiol.

[40] Guina T., Purvine S.O., Yi E.C., Eng J., Goodlett D.R., Aebersold R. (2003). Quantitative proteomic analysis indicates increased synthesis of a quinolone by *Pseudomonas aeruginosa* isolates from cystic fibrosis airways. Proc Natl Acad Sci U S A.

[41] Collier D.N., Anderson L., McKnight S.L., Noah T.L., Knowles M., Boucher R. (2002). A bacterial cell to cell signal in the lungs of cystic fibrosis patients. FEMS Microbiol Lett.

[42] Fletcher M.P., Diggle S.P., Camara M., Williams P. (2007). Biosensor-based assays for PQS, HHQ and related 2-alkyl-4-quinolone quorum sensing signal molecules. Nat Protoc.

