# Supplementary material for: Diagnostic and prognostic significance of systemic alkyl quinolones for P. aeruginosa in cystic fibrosis: A longitudinal study
Source: J Cyst Fibros. 2017 Mar;16(2):230–8. doi: 10.1016/j.jcf.2016.10.005 (PMC5345566; doi:10.1016/j.jcf.2016.10.005)
Supplement: Supplementary file 1 — Supplementary material [file mmc1.docx]

**Online data supplement for “Diagnostic and prognostic significance of systemic alkyl quinolones for *P. aeruginosa* in cystic fibrosis: a longitudinal study”**

**E1. Sample preparation**

Extracts of sputum samples for LC-MS/MS analysis were prepared by solvent extraction. Up to 1.0 mL of 50% (v/v) sputum suspension was spiked with 10 µL of an internal standard mix (1.0 µmol/L solution of deuterated PQS (PQS-d_4_) and extracted in triplicate with 0.5 mL volumes of 0.01% (v/v) acetic acid in ethyl acetate. After the addition of acidified solvent the samples were vortex-mixed for approximately 1 min and centrifuged (3 min at 12,000 g) with the analytes of interest partitioning into the organic phase. The combined organic extracts were dried under vacuum. Urine and plasma samples were prepared by solid phase extraction (SPE). The SPE cartridges (Waters, Oasis hydrophilic-lipophilic balanced (HLB), 60 mg, reversed-phase sorbent extraction cartridges), were pre-conditioned with 3 mL of methanol followed by 3.0 mL of 1% (v/v) acetic acid. Urine (1.0 mL) and plasma (0.5 mL) samples, diluted and acidified with an equal volume of 1% (v/v) acetic acid, were spiked with 10 µL of PQS-d_4_ internal standard solution (1.0 µmol/L in methanol). After loading onto the SPE cartridges, they were washed with 2 x 3 mL of 30% (v/v) methanol. The retained extracts, were eluted from the cartridges with 1.5 mL of methanol, and then dried under vacuum. Dried extracted samples were re-dissolved in 50 µL of 0.1% (v/v) formic acid in methanol prior to LC-MS/MS analysis.

**Preparation of calibration and quality control (QC) standards**

For the production of urine and plasma matrix matched calibration samples, blank samples (1.0 mL of urine and 500 µL of plasma) from a healthy volunteer donor were spiked with 50 µL of a methanolic mix of all the AQ standards prepared at a range of concentrations (0, 5, 15, 30, 60 and 100 nmol/L), giving an overall calibration range of 0-5 nmol/L for urine samples and 0-10 nmol/L for plasma samples.

Quality control samples were prepared similarly, spiking blank samples with 50 µL of the analyte mix at 10 and 80 nmol/L, producing plasma QC samples of 1 and 8 nmol/L, 0.5 and 4 nmol/L for urine.

In the absence of blank sputa to spike with analytes and produce matrix matched calibration and QC samples, 1.0 mL aliquots of 0.9% (w/v) NaCl were used, spiking with 50 µL of methanolic analyte mix at 0, 5, 25, 50, 100, 200, 400, and 1000 nmol/L. QC samples were prepared at 75 and 800 nmol/L.

All calibration and QC samples were prepared in triplicate, extracted and prepared ready for LC-MS/MS analysis as described above.

**LC-MS/MS analysis**

LC-MS/MS analysis was conducted according to Ortori *et al* 2011([26](#_ENREF_26)) on a 4000 QTRAP hybrid triple-quadruple linear ion trap mass spectrometer in tandem with a Shimadzu series 10AD VP LC system. The method is described briefly here. 20 µL of the prepared sputum, urine and plasma extracted samples were injected into the LC-MS/MS instrument for analysis. The chromatographic separation was achieved using a Phenomonex Gemini C18 reversed phase column (3.0 µm internal diameter, 100 x 3.0 mm) with a mobile phase flow rate of 450 µL/min. Mobile phases consisted of aqueous 0.1% (v/v) formic acid (A) and 0.1% (v/v) formic acid in methanol (B). The binary gradient began initially at 10% B and ran isocratically for the first 1 min before increasing linearly to 99% B over 9 min. After a further 5 min at this composition, the gradient was returned to 10% B over the next 1 min and allowed to re-equilibrate for 4 min. The MS, operating in the positive electrospray (+ES) mode, was set up for multiple reaction monitoring (MRM) to constantly screen the eluent from the LC column for all the analytes of interest.

**Sample quantification**

For each analyte, ratios of LC-MS/MS peak areas to internal standard peak areas were calculated and used to construct calibration lines of peak area ratio against analyte concentration. Results from the QC samples were used to ensure suitable precision and accuracy at both the high end and low end of the calibration lines. The lower limit of quantification (LLOQ) was established by using serial dilutions of the analyte mix and spiking into blank urine and plasma samples prior to extraction and analysis. The LLOQ was defined as the analyte concentration at which a signal/noise ratio of 10:1 was achieved.

For each analyte, linearity over the calibration ranges used was demonstrated. Results for the QC samples confirmed that analytical precision was <15% (<20% for the low end QC samples), and accuracy was 100±15% (100±20% for the low end QC samples), values that are generally considered acceptable for such analytical methodology. Calculated LLOQs in plasma and urine samples were as follows: (plasma) HHQ, 10 pmol/L; NHQ, 10 pmol/L; HQNO, 30 pmol/L; NQNO, 40 pmol/L; PQS, 100 pmol/L; C9-PQS, 100 pmol/L; and (urine) HHQ, 20 pmol/L; NHQ, 10 pmol/L; HQNO, 30 pmol/L; NQNO, 50 pmol/L; PQS, 50 pmol/L; C9-PQS, 50 pmol/L.

**E2. Correlations between quorum sensing signal molecule concentrations detected in sputum, plasma and urine in adults with cystic fibrosis at clinical stability.**

| **AQ** | **Sputum and Plasma**  **n=87** | **Sputum and Urine**  **n=88** | **Plasma and Urine**  **n=172** |
| --- | --- | --- | --- |
| HHQ | 0.80  <0.0001 | 0.79  <0.0001 | 0.76  <0.0001 |
| NHQ | 0.53  <0.0001 | 0.54  <0.0001 | 0.27  0.0004 |
| HQNO | 0.84  <0.0001 | 0.73  <0.0001 | 0.79  <0.0001 |
| NQNO | 0.75  <0.0001 | 0.45  <0.0001 | 0.58  <0.0001 |
| C7-PQS | 0.60  <0.0001 | 0.36  0.0005 | 0.58  <0.0001 |
| C9-PQS | 0.37  0.0004 | 0.30  0.004 | 0.19  0.014 |

Values are Spearman rank correlation co-efficients (above) with corresponding p values (below).

AQ = 2-alkyl-4-quinolone

HHQ = 2-heptyl-4-hydroxyquinoline

NHQ = 2-nonyl-4-hydroxyquinoline

HQNO = 2-heptyl-4-hydroxyquinoline-*N*-oxide

NQNO = 2-nonyl-4-hydroxyquinoline-*N*-oxide

C7-PQS = 2-heptyl-3-hydroxy-4(1*H*)-quinolone

C9-PQS = 2-nonyl-3-hydroxy-4(1*H*)-quinolone

n = number of participants with samples available for analysis

**E3. Sensitivity and specificity results for AQs in sputum, plasma and urine of adults with cystic fibrosis compared to hospital microbiological culture results.***

| **AQ** | **Sensitivity, %**  **(95% CI)** | **Specificity, %**  **(95% CI)** |
| --- | --- | --- |
| Sputum n=88 | | |
| HHQ | 71  (65-84) | 86  65-97) |
| NHQ | 73  (60-83) | 82  (60-95) |
| HQNO | 82  (70-70) | 82  (60-95) |
| NQNO | 77  (67-87) | 82  (60-95) |
| C7-PQS | 62  (49-74) | 68  (45-86) |
| C9-PQS | 70  (57-80) | 73  (50-89) |
| Plasma n=171 | | |
| HHQ | 62  (51-73) | 80  (70-88) |
| NHQ | 29  (20-40) | 88  (79-94) |
| HQNO | 48  (36-59) | 98  (92-100) |
| NQNO | 35  (25 -47) | 99  (94-100) |
| C7-PQS | 33  (23-44) | 99  (94-100) |
| C9-PQS | 10  (4-18) | 100  (96-100) |
| Urine n=173 | | |
| HHQ | 74  (63-83) | 84  75-91) |
| NHQ | 45  (34-57 | 77  (67-86) |
| HQNO | 49  (38-60) | 98  (92-100) |
| NQNO | 13  (7-22) | 98  (92-100) |
| C7-PQS | 37  (27-48) | 96  (89-99) |
| C9-PQS | 10  (4-20) | 97  (91-99) |

*Prevalence of *P. aeruginosa* using respiratory culture results was 75% in adults who provided spontaneous sputum samples, 48% in adults who provided plasma samples and 49% in adults who provided urine samples.

AQ = 2-alkyl-4-quinolone

HHQ = 2-heptyl-4-hydroxyquinoline

NHQ = 2-nonyl-4-hydroxyquinoline

HQNO = 2-heptyl-4-hydroxyquinoline-*N*-oxide

NQNO = 2-nonyl-4-hydroxyquinoline-*N*-oxide

C7-PQS = 2-heptyl-3-hydroxy-4(1*H*)-quinolone

C9-PQS = 2-nonyl-3-hydroxy-4(1*H*)-quinolone

A positive AQ test was defined as detectable signal above the lower limit of quantification in each media using LC-MS/MS (see online supplement S1).

**E4. Sensitivity and specificity results for AQs in plasma and urine of children with cystic fibrosis compared to hospital microbiological culture results.***

|  | **Sensitivity, %**  **(95% CI)** | **Specificity, %**  **(95% CI)** |
| --- | --- | --- |
| Plasma n=64 | | |
| HHQ | 86  (57-98) | 86  (73-94) |
| NHQ | 36  (13-65) | 96  (86-100) |
| HQNO | 57  (29-82) | 98  (89-100) |
| NQNO | 43  (18-71) | 98  (89-100) |
| C7-PQS | 43  (18-71) | 100  (89-100) |
| C9-PQS | 29  (8-58) | 98  (89-100) |
| Urine n=55 | | |
| HHQ | 79  (49-95) | 71  (55-84) |
| NHQ | 57  (29-82) | 73  (57-86) |
| HQNO | 71  (42-92) | 93  (80-99) |
| NQNO | 29  (8-58) | 95  (84-99) |
| C7-PQS | 50  (23-77) | 90  77-97) |
| C9-PQS | 29  (8-58) | 90  (77-97) |

*Prevalence of *P. aeruginosa* using respiratory culture results was 22% in children who provided plasma samples and 25% in children who provided urine samples.

A positive AQ test was defined as detectable signal above the lower limit of quantification in each media using LC-MS/MS.

AQ = 2-alkyl-4-quinolone

HHQ = 2-heptyl-4-hydroxyquinoline

NHQ = 2-nonyl-4-hydroxyquinoline

HQNO = 2-heptyl-4-hydroxyquinoline-*N*-oxide

NQNO = 2-nonyl-4-hydroxyquinoline-*N*-oxide

C7-PQS = 2-heptyl-3-hydroxy-4(1*H*)-quinolone

C9-PQS = 2-nonyl-3-hydroxy-4(1*H*)-quinolone

n = number of participants with samples available for analysis

**E5. Cross sectional AQ results for adults and children who had a new isolation of *P. aeruginosa* at the baseline visit, who were previously categorised as ‘free’** **from *P. aeruginosa****** **in the previous 12 months.**

| Patient details | Plasma AQs detected (pmol/L) | Urinary AQs detected (pmol/L) |
| --- | --- | --- |
| Child, PA free | HHQ (508), HQNO (157), NQNO (215), C7-PQS (150), C9-PQS (42) | HHQ (7639), NHQ (244), HQNO (4224), NQNO (141), C7-PQS (1175), C9-PQS (112) |
| Adult,  PA free | HHQ (36) | NHQ (41), HQNO (57) |
| Adult,  PA free | HHQ (25) | 0 |

**P. aeruginosa* status was classified using the Leeds criteria ([30](#_ENREF_30))

A positive AQ test was defined as detectable signal above the lower limit of quantification in each media using LC-MS/MS.

PA= *Pseudomonas aeruginosa*

AQ = 2-alkyl-4-quinolone

HHQ = 2-heptyl-4-hydroxyquinoline

NHQ = 2-nonyl-4-hydroxyquinoline

HQNO = 2-heptyl-4-hydroxyquinoline-*N*-oxide

NQNO = 2-nonyl-4-hydroxyquinoline-*N*-oxide

C7-PQS = 2-heptyl-3-hydroxy-4(1*H*)-quinolone

C9-PQS = 2-nonyl-3-hydroxy-4(1*H*)-quinolone

pmol/L = picomoles per litre

**E6. Prevalence^ of HHQ and HQNO in each media stratified by *P. aeruginosa* status^+^ in adults and children with CF and adult controls.**

|  | **PA status^+^** | **Sputum** | | **Plasma** | | **Urine** | |
| --- | --- | --- | --- | --- | --- | --- | --- |
|  |  | **HHQ**  % of positive samples  (total n) | **HQNO**  % of positive samples  (total n) | **HHQ**  % positive samples  (total n) | **HQNO**  % positive samples  (total n) | **HHQ**  % positive samples  (total n) | **HQNO**  % positive samples  (total n) |
| **Adults** | **Never** | 0  (3) | 0  (3) | 25  (20) | 0  (20) | 10  (20) | 0  (20) |
|  | **Free** | 20  (5) | 20  (5) | 18  (38) | 0  (38) | 3  (39) | 0  (39) |
|  | **Intermittent** | 0  (6) | 17  (6) | 27  (30) | 7  (30) | 31  (29) | 14  (29) |
|  | **Chronic** | 66  (74) | 76  (74) | 60  (84) | 48  (84) | 77  (86) | 47  (86) |
|  | **Controls** | - | - | 9  (22) | 5  (22) | 11  (19) | 0  (19) |
| **Children** | **Never** | - | - | 11  (18) | 0  (18) | 29  (14) | 0  (14) |
|  | **Free** | - | - | 13  (24) | 0  (24) | 18  (17) | 0  (17) |
|  | **Intermittent** | - | - | 36  (11) | 18  (11) | 46  (13) | 31  (13) |
|  | **Chronic** | - | - | 91  (11) | 64  (11) | 91  (11) | 82  (11) |

n= total number of samples analysed in each group

- no samples analysed

^ A positive sample for an alkyl quinolone was defined as a concentration ≥ the lower limit of quantification in each media using LC-MS/MS.

**^+^***P. aeruginosa* status was classified using the Leeds criteria ([30](#_ENREF_30))

PA = *P. aeruginosa*

HHQ = 2-heptyl-4-hydroxyquinoline

HQNO =2-heptyl-4-hydroxyquinoline-*N*-oxide

| Participants | Age (yrs), gender and *P. aeruginosa* status* for each patient at baseline | Plasma AQs detected  (concentration in pmol/L) | Urinary AQs detected  (concentration in pmol/L) |
| --- | --- | --- | --- |
| Adults | 25.6, F, Free | HHQ (29), NHQ (112) | NHQ (1518) |
|  | 24.6, M, Free | 0 | 0 |
|  | 33.9, M, Never | HHQ (36) | HHQ (47) |
|  | 29.6 , F, Never | HHQ (35) | 0 |
|  | 28.4, F, Free | 0 | 0 |
|  | 40.9, F, Free | HHQ (32) | NHQ (23) |
|  | 19.8, M, Free | 0 | 0 |
|  | 28.9, M, Free | HHQ (31) | NHQ (182) |
|  | 36.0, M, Free | 0 | 0 |
|  | 34.0, F, Free | 0 | 0 |
| Children | 3. 8, F, Free | HHQ (127), NHQ (185), NQNO (55) | − |
|  | 0.9, M, Never | HHQ (184) | 0 |
|  | 4.8, F, Free | 0 | NHQ (31) |
|  | 0.9, F, Never | HHQ (153) | − |
|  | 15.8, M, Free | 0 | 0 |
|  | 11.9, F, Free | HHQ (53) | NHQ (31) |

**E7. Longitudinal analysis: AQ results for 10 adults and 6 children with cystic fibrosis who were ‘free’ or ‘never’ having cultured *P. aeruginosa* cultured at baseline and subsequently became culture positive in the following year.**

**P. aeruginosa* status was classified using the Leeds criteria ([30](#_ENREF_30))

AQ = 2-alkyl-4-quinolone

HHQ = 2-heptyl-4-hydroxyquinoline

NHQ = 2-nonyl-4-hydroxyquinoline

HQNO = 2-heptyl-4-hydroxyquinoline-*N*-oxide

NQNO = 2-nonyl-4-hydroxyquinoline-*N*-oxide

C7-PQS = 2-heptyl-3-hydroxy-4(1*H*)-quinolone

C9-PQS = 2-nonyl-3-hydroxy-4(1*H*)-quinolone

− = no sample available for analysis

pmol/L = picomoles per litre

F= female

M= male
